# Retrospective analysis of inflammatory biomarkers and prognosis in non-small cell lung cancer without adenocarcinoma in situ

**DOI:** 10.3389/fgene.2025.1549602

**Published:** 2025-03-18

**Authors:** Qing Zhao, Songping Cui, Bin Hu, Shuo Chen

**Affiliations:** Beijing Chaoyang Hospital, Capital Medical University, Beijing, China

**Keywords:** NSCLC, inflammatory, biomarker, prognostic biomarker, lung cancer

## Abstract

**Background:**

Inflammatory biomarkers have shown prognostic value in Non-Small Cell Lung Cancer (NSCLC), but the inclusion of Adenocarcinoma *In Situ* (AIS) cases in previous studies may introduce bias. This study aims to evaluate the prognostic significance of inflammatory biomarkers in NSCLC while excluding AIS.

**Methods:**

This study included patients who received surgery for lung carcinoma from August 2016 and August 2019. We collected demographic, clinical, laboratory, and outcome information. Inflammatory biomarkers were analyzed using receiver operating characteristic (ROC) curves, Kaplan-Meier survival analysis, and Cox regression to assess their prognostic value.

**Results:**

Higher levels of inflammatory biomarkers correlated with poorer survival, with significant differences in overall survival (OS) between high- and low-expression groups. However, multivariate Cox regression identified age, tumor stage, and differentiation as independent prognostic factors, while biomarkers were not independently predictive.

**Conclusion:**

Inflammatory biomarkers have short-term prognostic value in invasive NSCLC, but traditional clinical and pathological factors remain key for long-term outcomes.

## Introduction

Lung cancer remains one of the most common and lethal malignancies worldwide. According to global cancer statistics, approximately 1.8 million deaths from lung cancer occur annually (Leiter et al., 2023). Data from the National Cancer Center of China indicate that in 2022, 733,000 lung cancer-related deaths ranked first among cancer-related deaths in the country, surpassing the combined deaths from the second and third most common cancers (Han et al., 2024). Despite the widespread application of low-dose computed tomography (LDCT), which has improved early detection rates and increased surgical opportunities for lung cancer patients, the overall long-term survival rate of lung cancer patients remains unsatisfactory.

Inflammation plays a critical role in the tumor microenvironment (TME). Tumor-associated neutrophils secrete cytokines that promote tumor angiogenesis and growth, while tumor-associated macrophages derived from monocytes facilitate immune escape and systemic immune dysregulation. Thus, inflammation-related clinical biomarkers, such as the systemic immune-inflammation index (SII), neutrophil-to-lymphocyte ratio (NLR), platelet-to-lymphocyte ratio (PLR), monocyte-to-lymphocyte ratio (MLR), and systemic inflammation response index (SIRI), may have prognostic value in cancer patients. Several clinical cohort studies have demonstrated the prognostic relevance of these inflammatory markers in various malignancies, including lung cancer (Templeton et al., 2014; Feng et al., 2020; Xie et al., 2023).

In China, non-small cell lung cancer (NSCLC) accounts for approximately 80% of all lung cancer cases, with adenocarcinoma and squamous cell carcinoma being the predominant histological subtypes. In recent years, the incidence of adenocarcinoma *in situ* (AIS), primarily characterized by ground-glass opacities (GGOs) on imaging, has increased significantly. As a malignancy without histological invasion, AIS has a highly favorable long-term prognosis (Inamura, 2018). However, many predictive models for lung cancer have not explicitly excluded AIS patients, potentially introducing confounding factors into their findings.

This study aims to explore prognostic factors for NSCLC patients after excluding AIS cases to construct a more precise inflammation-related survival prediction model. The findings may provide a reference for the rapid and convenient application of these markers in clinical practice.

## Materials and methods

### Study population

**FIGURE 1:**
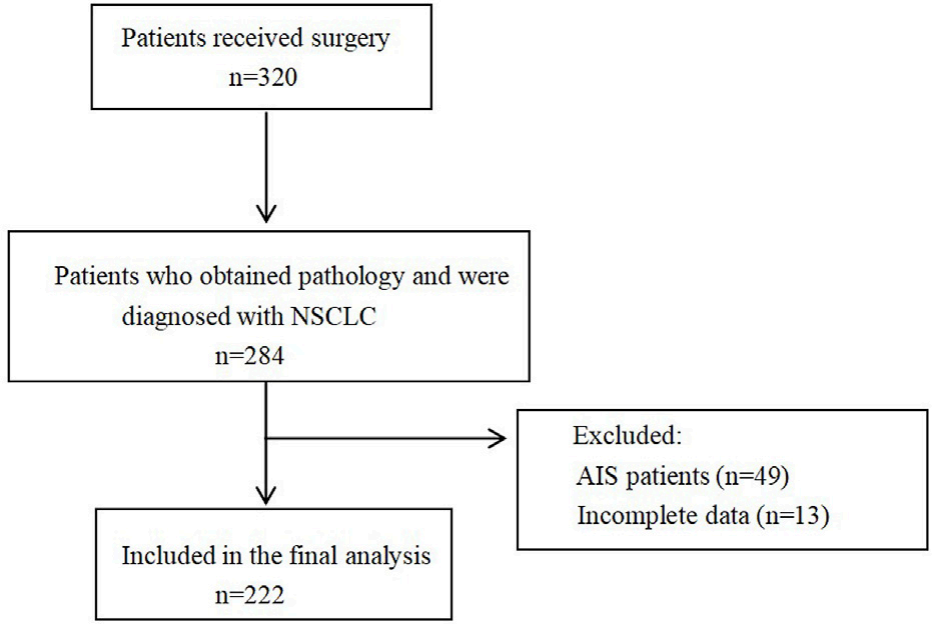
The Inclusion and Exclusion Flowchart of the study cohort.

This retrospective cohort study included NSCLC patients who underwent surgical treatment at Beijing Chaoyang Hospital between August 2016 and August 2019. Patients with *in situ* lung cancer were excluded to ensure a homogeneous study population. The final cohort consisted of 222 patients with complete clinical, pathological, and survival data. The selection of the study population is represented by a flowchart (Figure 1).

### Data collection and follow-up

We collected the following data using an electronic medical record system: patient baseline information (age, gender, smoking history, and family history, etc.), laboratory test results (complete blood count, etc.), surgical-related data (surgery time, intraoperative bleeding volume, etc.) and pathological results (pathological type, degree of differentiation, etc.). All patients who were enrolled had venous blood samples collected within 24 h of admission and underwent a complete blood count analysis. All hematology was collected prior to the patient’s surgery. In this study, inflammatory biomarkers (MLR, PLR, NLR, SII, and SIRI) were derived from complete blood count (CBC) tests. All blood samples were collected within 24 h of patient admission. The samples were analyzed using an automated hematology analyzer. The biomarkers were calculated as follows: MLR (Monocyte-to-Lymphocyte Ratio) was calculated by dividing the monocyte count by the lymphocyte count; NLR (Neutrophil-to-Lymphocyte Ratio) was calculated by dividing the neutrophil count by the lymphocyte count; PLR (Platelet-to-Lymphocyte Ratio) was calculated by dividing the platelet count by the lymphocyte count; SII (Systemic Immune-Inflammation Index) was calculated by multiplying the neutrophil count by the platelet count and dividing by the lymphocyte count; SIRI (Systemic Inflammation Response Index) was calculated by multiplying the monocyte count by the neutrophil count and dividing by the lymphocyte count.

### Statistical analysis

Continuous variables were compared between groups by either the Student’s t-test or the Mann-Whitney U-test. Categorical variables were compared between groups by Pearson’s chi-square test or Fisher’s exact test. Time-dependent receiver operating characteristic (ROC) curves were utilized to determine optimal cutoff values for the biomarkers in predicting 1,3 and 5-year overall survival (OS). Corresponding area under the curve (AUC) values were calculated, and patients were categorized into high- and low-expression groups based on these thresholds. OS was defined as the interval from surgery to death or last follow-up. Kaplan–Meier curves with the log-rank test were used to survival analysis. Univariate Cox regression identified potential prognostic factors. Variables with P < 0.05 were included in multivariate Cox regression. Statistical significance was defined as two-sided P < 0.05. All statistical analyses were performed using SPSS 26.0 and GraphPad Prism 8.0.

## Results

### Characteristics of patients

A total of 222 patients diagnosed with NSCLC who underwent surgical treatment were included in this study. The mean age of the cohort was 63 months, comprising 133 male and 89 female patients. The median follow-up duration was 77 months. Patients were stratified into groups based on survival outcomes.

Baseline characteristics are summarized in Table 1. Significant differences were observed between the survival and non-survival groups in terms of sex, age, and smoking history. However, no significant differences were found in BMI, hypertension, diabetes, or coronary artery disease. Regarding surgical variables, differences in hospitalization duration, surgical approach, and operation time were statistically significant. Pathological characteristics, including tumor differentiation, histological type, vascular invasion, and tumor thrombus, also showed significant differences between the groups. Among inflammatory markers, preoperative white blood cell, neutrophil, and monocyte counts exhibited significant differences, as did the derived markers NLR, MLR, PLR, SII, and SIRI.

**TABLE 1:** Demographic and baseline characteristics of the total cohort.

Variables	Total (n = 222)	Survival (n = 122)	Non-Survival (n = 100)	P-Value
Age	61.4±9.5	60.1±9.4	63.1±9.5	0.02
Gender(male)	133(59.9)	59(48.4)	74(74.0)	<0.001
BMI	24.0±3.5	24.0±3.4	24.0±3.7	0.742
Length of Stay (days)	13.0(6.0)	13.0(4.3)	13.0(7.0)	0.012
Surgical approach				<0.001
VATS	157(70.7)	102(83.6)	55(55.0)	
Open thoracic surgery	65(29.3)	20(16.4)	45(45.0)	
Surgical time (min)	180.0(60.0)	175(46.3)	180(87.5)	0.006
Blood loss (ml)	150.0(100.0)	100.0(100.0)	150.0(200.0)	0.219
Severe pulmonary disease	18(8.1)	8(6.6)	10(10.0)	0.352
Hypertension	65(29.3)	33(27.0)	32(32.0)	0.422
Coronary heart disease	19(8.6)	8(6.6)	11(11.0)	0.241
Diabetes	24(10.8)	14(11.5)	10(10.0)	0.726
Smoking history	105(47.3)	46(37.7)	59(59.0)	0.001
DVT	33(14.9)	18(14.8)	15(15.0)	0.959
Clinical Staging				<0.001
I	119(53.6)	91(75.4)	28(28.0)	
II	50(22.5)	18(14.8)	32(32.0)	
III	52(23.4)	12(9.8)	40(40.0)	
Degree of differentiation				<0.001
Low	47(21.2)	18(14.8)	29(29.0)	
Medium	131(59.0)	69(56.6)	62(62.0)	
High	42(18.9)	34(27.9)	8(8.0)	
Pathological type				<0.001
ADC	153(68.9)	97(79.5)	56(56.0)	
SC	69(31.1)	25(20.5)	44(44.0)	
Vascular invasion	61(27.5)	23(18.9)	38(38.0)	0.001
Vascular tumor thrombus	35(15.8)	11(9.0)	24(24.0)	0.002
White blood cell (×109/L)	6.40±2.16	5.90±1.57	7.01±2.59	<0.001
Neutrophil (×109/L)	4.00±1.91	3.54±1.35	4.55±2.30	<0.001
Lymphocyte (×109/L)	1.90±1.54	1.80±0.57	2.01±2.19	0.326
Monocyte (×109/L)	0.42±0.44	0.36±0.14	0.48±0.63	0.036
Red blood cell (×109/L)	4.48±0.47	4.53±0.45	4.41±0.49	0.06
Albumin (g/L)	138.0±15.2	139.1±14.6	136.8±15.9	0.26
PLT	239.9±68.3	236.7±68.5	243.7±68.3	0.45
NLR	2.10(1.40)	1.97(1.07)	2.36(1.63)	0.002
PLR	130.2(71.9)	127.8(61.1)	137.8(92.8)	0.088
MLR	0.21(0.13)	0.20(0.10)	0.23(0.15)	0.002
SII	464.3(390.7)	426.0(315.0)	557.1(547.2)	0.002
SIRI	0.72(0.71)	0.66(0.49)	0.90(1.08)	<0.001

DVT, Deep vein thrombosis; SC, Squamous cell carcinoma; ADC, Adenocarcinoma

### Preoperative inflammatory biomarkers and prognosis

**FIGURE 2:**
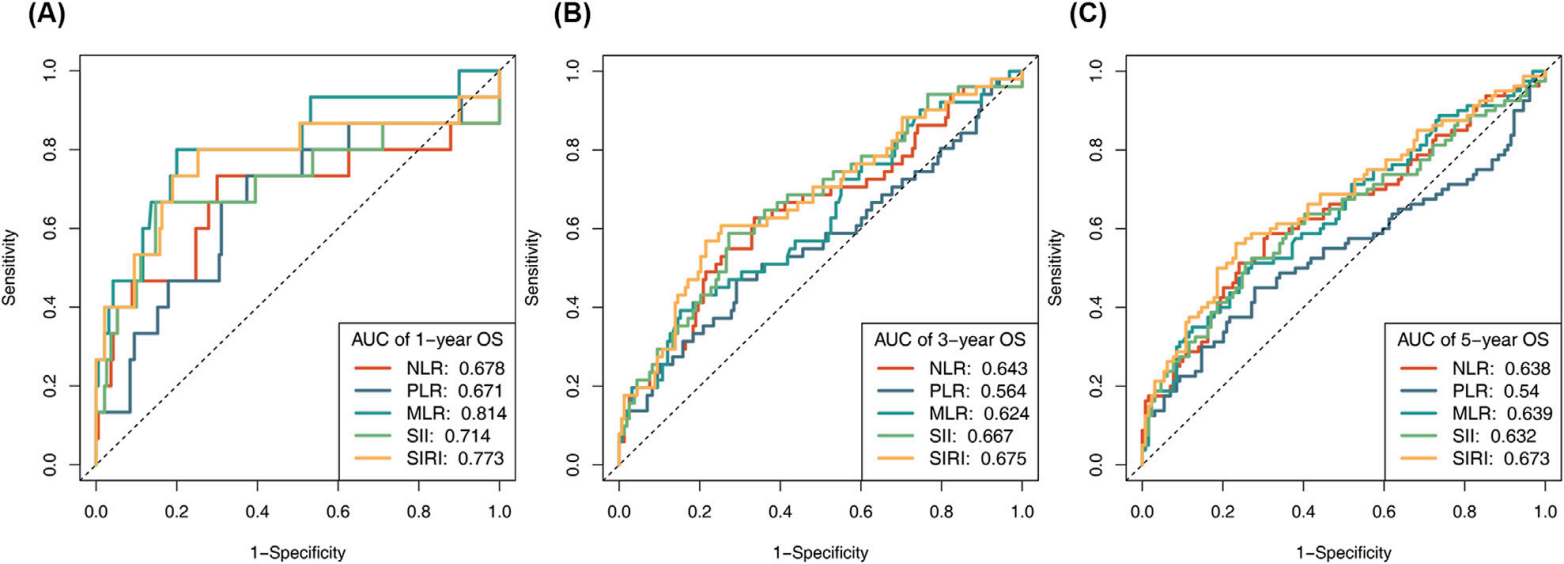
The receiver operating characteristic (ROC) curves explore the value of NLR, PLR, MLR, SII and SIRI in predicting **(A)** 1-year survival, **(B)** 3-year survival, and **(C)** 5-year survival outcomes of NSCLC patients.

Time-dependent ROC curves were employed to investigate the correlation between preoperative inflammatory biomarkers and postoperative prognosis (Figure 2). The optimal cutoff values for NLR, PLR, MLR, SII, and SIRI in predicting 1-year OS were determined, with corresponding AUCs of 0.741, 0.731, 0.756, 0.746, and 0.749, respectively. Based on these cutoff values, patients were divided into high- and low-expression groups, and Cox regression analysis was performed.

**FIGURE 3:**
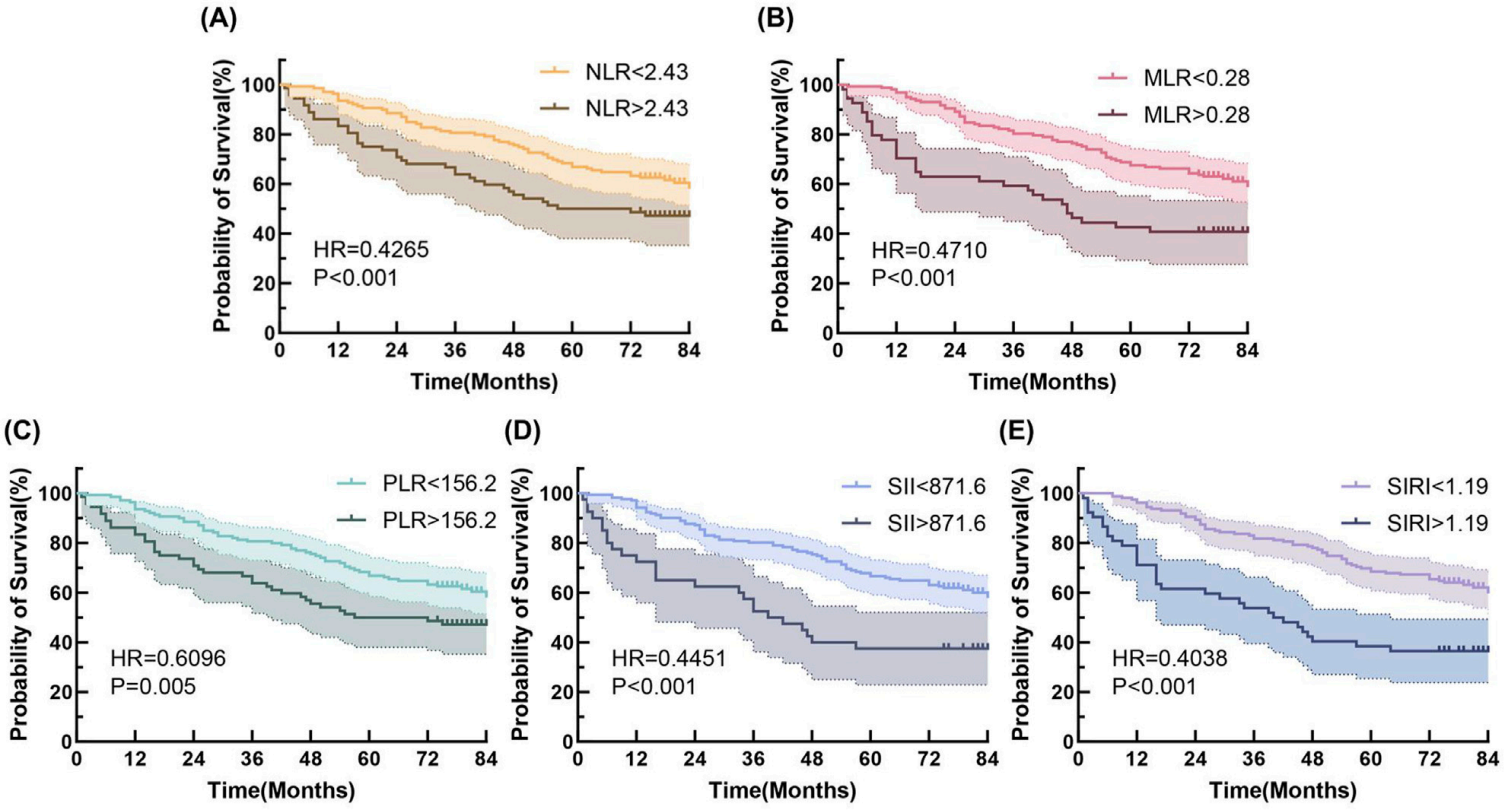
Kaplan-Meier curves for long time prognosis by **(A)** NLR, **(B)** MLR, **(C)** PLR, **(D)** SII and **(E)** SIRI.

Univariate Cox regression analysis results are presented in Table 2. Variables with statistical significance were included in multivariate Cox regression analysis, which revealed that age, operation time, tumor stage, and differentiation were independent factors associated with OS. Long-term prognosis was assessed using Kaplan-Meier survival analysis (Figure 3). Patients were stratified into high- and low-expression groups based on the five inflammatory biomarkers. The low-expression groups consistently demonstrated significantly higher OS rates compared to the high-expression groups, with all group differences being statistically significant (log-rank test, p < 0.001).

**TABLE 2:** Cox regression examination investigating the impact of clinicopathological variables on patients’ overall survival.

Variables	Univariate Cox regression		Multivariate Cox regression	
	HR (95% CI)	P	HR (95% CI)	P
Gender(male)	0.420 (0.226-0.664)	<0.001	0.676 (0.370-1.237)	0.204
Age	1.030 (1.007-1.053)	0.010	1.043 (1.017-1.071)	0.001
Length of Stay (days)	1.050 (1.014-1.088)	0.006	0.998 (0.955-1.042)	0.925
Surgical approach	2.965 (1.973-4.457)	<0.001	1.626 (0.999-2.647)	0.050
Surgical time (min)	1.006 (1.002-1.010)	0.001	1.005 (1.000-1.009)	0.046
Smoking history	2.022 (1.340-3.050)	0.001	1.638 (0.959-2.797)	0.071
Clinical Staging	2.313 (1.831-2.922)	<0.001	1.778 (1.305-2.423)	<0.001
Degree of differentiation	0.477 (0.344-0.664)	<0.001	0.604 (0.411-0.886)	0.010
Pathological type	2.110 (1.402-3.175)	<0.001	0.663 (0.380-1.158)	0.148
Vascular invasion	1.968 (1.293-2.997)	0.002	1.213 (0.762-1.932)	0.415
Vascular tumor thrombus	1.992 (1.244-3.188)	0.004	1.138 (0.665-1.947)	0.638
NLR	2.354 (1.572-3.526)	<0.001	1.247 (0.679-2.290)	0.478
PLR	1.589 (1.053-2.396)	0.027	1.156 (0.683-1.955)	0.589
MLR	2.056 (1.347-3.139)	0.001	0.738 (0.393-1.383)	0.343
SII	2.209 (1.398-3.492)	0.001	1.084 (0.491-2.390)	0.842
SIRI	2.500 (1.641-3.809)	<0.001	1.767 (0.797-3.917)	0.161

## Discussion

Chronic inflammation has been widely recognized as a critical factor in cancer development and progression. Clinical studies examining inflammation-related prognostic biomarkers in lung cancer patients have proliferated in recent years. However, there remains a lack of long-term follow-up data from NSCLC cohorts that exclude AIS patients. In this retrospective study, we investigated prognostic factors in NSCLC patients after excluding AIS cases. Our findings confirmed significant differences in survival based on levels of various inflammatory biomarkers, with higher levels correlating with poorer long-term survival.

Inflammatory cells and their derived indices play pivotal roles in tumor initiation, progression, and metastasis (Inamura, 2018; Diakos et al., 2014). These indices reflect the complex interplay between pro-tumorigenic and immune surveillance dysregulation within the TME. For instance, tumor-associated neutrophils promote angiogenesis by secreting vascular endothelial growth factor (VEGF) and activating oncogenic pathways such as NF-κB via chronic reactive oxygen species (ROS) release. Tumor-associated macrophages (TAMs), particularly M2-polarized TAMs, secrete VEGF and matrix metalloproteinases (MMPs), fostering tumor angiogenesis and invasion (Zou et al., 2017; Swierczak et al., 2015; Barbetta et al., 2018; Cortese et al., 2020). Platelets contribute to tumor metastasis by facilitating epithelial-mesenchymal transition (EMT) and protecting circulating tumor cells (Żmigrodzka et al., 2020). These cellular processes are encapsulated by inflammatory indices such as NLR, PLR, MLR, SII, and SIRI (Qi et al., 2016). Numerous studies have demonstrated the prognostic value of these markers in various malignancies, including lung, liver, and breast cancers (Hu et al., 2014; Chen et al., 2020). For example, a follow-up study of 1,431 stage I lung adenocarcinoma patients found significant correlations between NLR, SII, SIRI, and cancer-specific survival (CSS) or disease-free survival (DFS) (Shen et al., 2021). Similarly, studies in advanced lung cancer cohorts have established associations between elevated SIRI levels and poorer prognosis, as well as similar findings for NLR and PLR (Jiang et al., 2021; Zuo et al., 2023).

However, our study found that while higher levels of these inflammatory markers were associated with poorer survival, they did not independently predict survival in multivariate Cox regression analyses. This is in contrast to some of the findings in the existing literature, where a number of studies have demonstrated the potential of various inflammatory markers to independently predict long-term survival in predicting lung cancer prognosis. For example, a summary evaluation of 86 meta-analyses on NLR and prognosis of malignant tumors showed that there is a strong association between elevated NLR and poor prognosis in cancer patients (Cupp et al., 2020). A study by Aguiar-Bujanda et al. found that SII had a high clinical value. Elevated SII was associated with advanced tumor stage and lymph node metastasis, and could be used as a prognostic predictor in patients with advanced tumors (Aguiar-Bujanda et al., 2018). This discrepancy may be attributed to potential interactions among these markers, diminishing their individual contributions in multivariate models. Additionally, inflammatory markers may primarily reflect short-term changes within the TME, while long-term prognostic factors such as tumor stage and pathological features overshadow their influence. Long-term prognosis is more influenced by the biological behavior of the tumor and postoperative treatment. Supporting this hypothesis, our time-dependent ROC analysis revealed higher diagnostic efficacy for 1-year survival compared to three- or 5-year survival endpoints. Kaplan-Meier survival analysis further demonstrated that patients with lower levels of NLR, PLR, MLR, SII, and SIRI exhibited significantly better survival outcomes. This underscores the potential utility of these markers in short-term prognosis, though their inclusion as independent factors in clinical prediction models remains limited.

Multivariate Cox regression identified age, operation time, tumor stage, and differentiation as independent prognostic factors for OS, consistent with previous studies. These findings emphasize the importance of clinical and pathological characteristics in NSCLC prognosis. Yotsukura’s team conducted clinical follow-up on 524 postoperative patients with pathological diagnosis of AIS, indicating that the tumor-related survival rate after surgery was nearly 100% (Jiang et al., 2021; Yotsukura et al., 2021). Our cohort excluded AIS patients, whose favorable prognosis and unique biological behavior differ markedly from invasive cancers. This exclusion enhanced the specificity of our results by directly linking prognosis to tumor-related characteristics rather than confounding factors such as localized inflammation or early-stage disease features.

Although this study systematically analyzed the prognostic impact of inflammatory biomarkers on NSCLC patients through long-term follow-up, certain limitations remain. First, as a single-center retrospective study, it may be subject to unavoidable selection bias. Second, the dynamic changes of inflammatory biomarkers were not included in the analysis, which might limit their predictive value in long-term follow-up. Despite these limitations, this study has notable clinical significance. By excluding AIS patients, we clarified the short-term prognostic value of inflammatory biomarkers in invasive NSCLC and reaffirmed the central role of traditional clinical and pathological features (such as tumor stage and differentiation) in long-term prognosis. While our study mainly focused on biomarkers, we recognize that inflammatory factors such as IL-6, TNF-α, and MCP-1 may also significantly influence the prognosis of NSCLC. IL-6 and TNF-α promote tumor progression by enhancing inflammation and creating an immunosuppressive microenvironment, while MCP-1 recruits monocytes and macrophages, potentially aiding immune evasion and metastasis. Although these factors were not measured in our study, we recommend that future research investigate their specific effects on tumor cells. Based on these findings, we propose that the application of inflammatory biomarkers in postoperative management of invasive lung cancer requires further confirmation through prospective studies.

## Conclusion

Inflammatory markers, while valuable in short-term prognostic evaluation, cannot yet be considered independent risk factors. Traditional clinical and pathological features (e.g., age, operation time, tumor stage, and differentiation) have clear independent prognostic value in NSCLC. Close monitoring of patients with high inflammatory marker levels is warranted and may provide clinical benefit in improving long-term outcomes.

## Data Availability

The raw data supporting the conclusions of this article will be made available by the authors, without undue reservation.
